# A157 PREDICTING SURVIVAL TIME FOR BOWEL RESECTION IN INDIVIDUAL CROHN DISEASE PATIENTS

**DOI:** 10.1093/jcag/gwae059.157

**Published:** 2025-02-10

**Authors:** R G Suarez Suarez, S Ahmed, H Huynh, A Shaikh, A Otley, K Jacobson, M Zachos, C Deslandres, W El-Matary, J deBruyn, A Griffiths, T Walters, E Wine

**Affiliations:** Paediatrics, University of Alberta, Edmonton, AB, Canada; Paediatrics, University of Alberta, Edmonton, AB, Canada; Paediatrics, University of Alberta, Edmonton, AB, Canada; The Hospital for Sick Children, Toronto, ON, Canada; Dalhousie University, Halifax, NS, Canada; BC Children’s Hospital, Vancouver, BC, Canada; McMaster University, Hamilton, ON, Canada; Universite de Montreal, Montreal, QC, Canada; University of Manitoba, Winnipeg, MB, Canada; University of Calgary, Calgary, AB, Canada; The Hospital for Sick Children, Toronto, ON, Canada; The Hospital for Sick Children, Toronto, ON, Canada; Paediatrics, University of Alberta, Edmonton, AB, Canada

## Abstract

**Background:**

Pediatric onset Crohn disease (pCD) tends to be more complicated with a higher likelihood of requiring intestinal resection surgery. Currently there are no predictive tools for surgery in pCD; risk scores and relative survival analysis are available, but these cannot accurately calculate the probability of surgery in children with CD at an individual level.

**Aims:**

This study aims to apply machine learning to create an individual survival time distributions (ISD) tool for accurate prediction of bowel resection over time in a novel pCD patient.

**Methods:**

A prospectively-followed cohort of pCD patients (n= 934) was collected through the Canadian Children Inflammatory Bowel Disease Network (CIDsCaNN) inception cohort (2013-2021). 58 of the 934 pCD patients underwent surgery, while the remaining 876 either did not require surgery by the study’s end, transitioned to adult care, or were lost to follow-up. Surgeries included bowel resections for stricturing or penetrating disease.

Clinical, laboratory, and treatment data were compiled into three datasets: baseline, induction (baseline + 10 weeks follow-up data), and longitudinal (baseline + 3 equally spaced follow-up visits with all treatment data).

To develop the ISD predictor, we built a learning algorithm that includes maximum relevance minimum redundancy (mRMR) feature selection, Cox Proportional Hazards, and Random Survival Forest models. To estimate the quality of the ISD predictor, measured by the Integrated Brier Score (IBS) and Concordance Index (C-Index), we used k-fold (external) cross validation.

**Results:**

Our resulted ISD predictors, two trained Cox Proportional Hazards models and one Random Survival Forest model, had IBS scores < 0.1 and C-Index scores > 0.83. These results indicate that our models provide a reliable ranking of survival times based on the individual probability for surgery. The results also showed that the features that are most informative for prediction of surgery were stricturing behavior, penetrating behavior, and distal bowel involvement.

**Conclusions:**

This study suggests that it is possible to produce an ISD predictor capable of forecasting bowel resection over future times in a novel pCD patient. This novel approach can build tools to improve both the choice and timing of therapy.

**Funding Agencies:**

CIHRWomen and Children’s Health Research Institute

